# Grain yield, adaptation and progress in breeding for early-maturing and heat-tolerant wheat lines in South Asia

**DOI:** 10.1016/j.fcr.2016.04.017

**Published:** 2016-06

**Authors:** S. Mondal, R.P. Singh, E.R. Mason, J. Huerta-Espino, E. Autrique, A.K. Joshi

**Affiliations:** a International Maize and Wheat Improvement Center (CIMMYT), Int. Apdo. Postal 6-641, 06600 Mexico, DF, Mexico; b Department of Crop, Soil and Environmental Science, University of Arkansas, 115 Plant Sciences Building, Fayetteville, AR 72701, USA; c Campo Experimental Valle de Mexico INIFAP, Apdo. Postal 10, 56230 Chapingo, Edo. de Mexico, Mexico; d International Maize and Wheat Improvement Center (CIMMYT), South Asia Regional Office, Singh Durbar Plaza Road, Kathmandu, Nepal; e Department of Genetics and Plant Breeding, Institute of Agricultural Sciences, Banaras Hindu University, Varanasi, India

**Keywords:** GY, grain yield, DTH, days to heading, DTM, days to maturity, PH, plant height, ME, mega environments, Early maturity, Wheat, Heat tolerance, South Asia

## Abstract

Maintaining wheat productivity under the increasing temperatures in South Asia is a challenge. We focused on developing early maturing wheat lines as an adaptive mechanism in regions suffering from terminal heat stress and those areas that require wheat adapted to shorter cycles under continual high temperature stress. We evaluated the grain yield performance of early-maturing heat-tolerant germplasm developed by CIMMYT, Mexico at diverse locations in South Asia from 2009 to 2014 and estimated the breeding progress for high-yielding and early-maturing heat-tolerant germplasm in South Asia. Each year the trial comprised of 28 new entries, one CIMMYT check (Baj) and a local check variety. Locations were classified by mega environment (ME); ME1 being the temperate irrigated locations with terminal high temperature stress, and ME5 as hot, sub-tropical, irrigated locations. Grain yield (GY), days to heading (DTH) and plant height (PH) were recorded at each location. Effect of temperature on GY was observed in both ME1 and ME5. Across years, mean minimum temperatures in ME1 and mean maximum temperatures in ME5 during grain filling had significant negative association with GY. The ME1 locations were cooler that those in ME5 in the 5 years of evaluations and had a 1–2 t/ha higher GY. A mean reduction of 20 days for DTH and 20 cm in PH was observed in ME5. Negative genetic correlations of −0.43 to −0.79 were observed between GY and DTH in South Asia during 2009–2014. Each year, we identified early-maturing germplasm with higher grain yield than the local checks. A positive trend was observed while estimating the breeding progress across five years for high-yielding early-maturing heat tolerant wheat compared to the local checks in South Asia. The results suggests the potential of the high-yielding early-maturing wheat lines developed at CIMMYT in improving wheat production and maintaining genetic gains in South Asia.

## Highlights

• Each year from 2009 to 2014, 28 newly developed early-maturing high-yielding CIMMYT wheat lines were evaluated across locations in South Asia.
• Maximum temperatures in ME5 (continual high temperature stress regions) and minimum temperature in ME1 (terminal high temperature stress regions) had significant impact on grain yield in South Asia.
• Significant negative genetic correlations of grain yield with days to heading.
• Early maturity has the potential to improve adaptation and maintenance of genetic gains in South Asia.

## 1 Introduction

Wheat, an important source of calories and proteins is a key cereal crop that impacts the global economy and food security. Continuous development of agronomically superior wheat varieties with high grain yield (GY), good nutrition and processing quality and tolerance to biotic and abiotic stresses is critical for ensuring food security. South Asia (comprised of India, Nepal, Pakistan and Bangladesh) is one of the most important wheat producing and consuming regions in the world. Though wheat production in South Asia has increased dramatically since the Green Revolution, multiple challenges such as high temperature stress and reduced water availability are major concerns. Rao et al. (2014) reported a rise of 0.32 °C and 0.28 °C per decade in the minimum and maximum temperatures over wheat growing areas in India. Warmer temperatures have already been determined to be one of the major factors in slowing the wheat productivity growth in South Asia and globally (Gourdji et al., 2013, Pask et al., 2014, Lobell et al., 2012, Sharma et al., 2007, Joshi et al., 2007a). Estimated GY losses in South Asia can range from 6 to 10% per °C rise in temperature during the grain-filling period (Lobell et al., 2008, Mondal et al., 2013, Asseng et al., 2015). Further, the current estimates by the World Bank indicate a population of 1.6 billion in South Asia, which is nearly 24% of the world population, adds to the urgency of increasing wheat production and maintaining food security.

Though a cool season crop, wheat is widely grown in temperate, tropical and subtropical areas of South Asia. The subtropical western Indo-Gangetic Plain of South Asia has a cool climate during the crop growing season and late incidence of high temperatures (>30 °C) during advanced grain filling. In contrast, the eastern, central and southern regions of South Asia are warmer throughout the crop season with maximum temperature ranges of 27–30 °C during the vegetative stages that gradually rises above 30 °C during grain filling. Thus, there is demand for prioritization of developing new wheat varieties with improved heat tolerance in South Asia.

The Cereal Systems Initiative in South Asia (CSISA), a collaborative effort between CGIAR centers (CIMMYT, IRRI, IFPRI, and ILRI) and national programs was established in 2009 to improve cereal productivity in South Asia (http://csisa.org). The CIMMYT bread wheat breeding program focused on developing early-maturing and heat tolerant wheat lines. Early maturity to escape high temperature stress has been suggested is an excellent crop adaptation approach in regions suffering from terminal and continual high temperature stress (Joshi et al., 2007b, Mondal et al., 2013). The new approach in breeding for early maturity has led to distribution and evaluation of trials in diverse locations in South Asia since 2009. CIMMYT wheat germplasm has shown excellent adaption to a wide range of climates and has been either directly released or been an ancestor of wheat varieties globally (Singh et al., 2007) and genetic gains have been reported in both optimal and stressed environments (Singh et al., 2007, Gourdji et al., 2012, Manes et al., 2012).

Our objectives were to evaluate the performance of early-maturing heat-tolerant germplasm developed in Mexico at diverse locations in South Asia from 2009 to 2014 and to estimate the breeding progress in developing high-yielding and early-maturing heat-tolerant germplasm for South Asia.

## 2 Materials and methods

### 2.1 Trial locations and climate data

Each year since 2009, high-yielding, early-maturing, heat-tolerant wheat genotypes were selected from advanced yield trials conducted at the Norman E. Borlaug Experiment Station (CENEB) in Ciudad (Cd.) Obregon, Sonora, Mexico (latitude 27.33, longitude −109.93, 40 msal). The CIMMYT advanced yield trials are tested across multiple environments in Cd. Obregon. As part of CSISA, the advanced lines with stable grain yields under irrigated normal and late sown (for high temperature stress) environments constituted the CSISA Heat Tolerant Early Maturity Yield Trial (CSISA-HT-EM). These trials were evaluated in collaboration with national program partners across several locations in major wheat producing regions of Bangladesh, India, Nepal, and Pakistan from 2009 to 2014 for GY performance and adaptation (Table 1). Each CSISA-HT-EM trial included 28 new entries, one CIMMYT check variety (Baj), and one local check, i.e., the best locally adapted variety at each location. Each trial had 3 replicates and was arranged in an alpha lattice design. Information on locations, sowing and harvest dates and plot sizes are presented in Table 1. Management practices were based on the established procedures followed at each individual location which are similar to those used for national yield trials conducted at that location. In South Asia, wheat is sown in November/December and harvested in March/May of the following year, depending on the location.

Locations in South Asia were also classified into mega environments (ME) based on the CIMMYT classification system described by Rajaram et al. (1995) and Braun et al. (2010), with ME1 and ME5 being most relevant to the studied region. This classification system defines ME1 as an optimally irrigated and highly productive environment where wheat grows in cool temperature but suffers from terminal heat stress and ME5 as hot, humid or non-humid, tropical, or subtropical regions, with continuous high temperatures during the crop season and the mean temperatures in the coolest month is >17.5 °C. These two MEs can be further differentiated based on the mean minimum temperature ranges of the coolest quarter, 3–11 °C for ME1 and 11–16 °C for ME5 (Ortiz et al., 2008).

Consistent weather data was not available for all locations across years. Thus mean temperature data during the crop season was extrapolated from NASA POWER Data (NASA, 2016) for the following locations during 2009–2014: Dinajpur and Jessore in Bangladesh, Karnal, Indore, Ludhiana, New Delhi, Ugar, Varanasi, Jabalpur in India, Bhairahawa in Nepal and Faisalabad in Pakistan. The maximum and minimum temperatures for the some of the same locations were either received from the collaborator or extracted from online archived weather data (www.wunderground.com).

### 2.2 Grain yield and agronomic traits

At the end of the crop season, collaborators provided data on GY (t/ha), days to heading (DTH), days to maturity (DTM), plant height (PH) and trial management practices. DTH was estimated as the number of days from sowing date/first irrigation till 50% of the spikes had emerged from the flag leaf. DTM was recorded as senescence in the peduncles of 50% of the spikes. At maturity, plots were harvested to determine GY.

### 2.3 Statistical analysis

Data for GY and agronomic traits for each trial were analyzed by using a mixed model for computing the least square means (LSMEANS) for each genotype at individual locations and across locations and MEs in each year using the program Multi Environment Trial Analysis with R for Windows (METAR, Alvarado et al., 2015). Genetic correlations between GY and DH were also estimated using METAR. The Dunnett’s (one-tail) test and Fisher’s LSD were estimated to compare the mean grain yield of the lines. The estimated LSMEANS of GY for each genotype was expressed as a percentage of the local check using the following formula:

$$\%GY=\left(\frac{GY_{g}}{GY_{c}}\right)\times 100$$

where, $GY_{g}$ is the mean GY of a genotype and $GY_{c}$ is the mean GY of the local check.

Broad sense heritability (H) was estimated for each trait in the multi environment trial planted in *e* environment using the following formula:

$$H=\frac{\sigma_{g}^{2}}{\sigma_{g}^{2}+\sigma_{ge}^{2}/e+\sigma_{e}^{2}/er}$$

where, $\sigma_{g}^{2}$ is the genetic variance, $\sigma_{e}^{2}$ is the residual variance, $\sigma_{ge}^{2}$ is genotype x environment (or location) interaction variance, *e* is the number of environments/locations and *r* is the number of replicates.

Regression analysis was performed to measure the rate of progress in breeding for early-maturing high-yielding heat tolerant wheat lines (Sayre et al., 1997, Sharma et al., 2012). The mean%GY of the five highest yielding lines (HYL) over the local checks was regressed over the 5 years of evaluations and the rate of progress was estimated from the slope of the regression line.

## 3 Results

### 3.1 Location distribution, classification and climate

The CSISA-HT-EM trials were evaluated in a diverse set of locations across the major wheat producing areas of South Asia with India having the largest number of locations each year (Table 1, Supplementary Table 1). The sowing dates ranged from 1st week of November till last week of December. The plot sizes and management practices varied between locations depending on the local practices followed by the National Partners. Individual locations were also classified into MEs (Table 2). Both ME1 and ME5 included irrigated environments, though the nature of high temperature stress varies; ME1 locations experience terminal high temperature stress; ME5 locations suffer from continual high temperatures during wheat growing season.

**Fig. 1:**
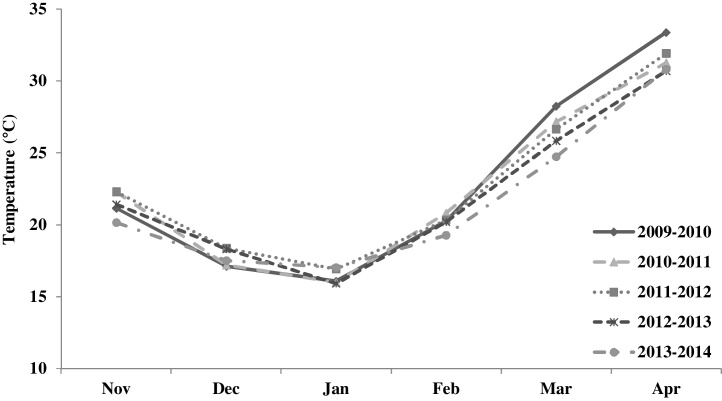
Mean monthly temperatures from November to April in five years (2009–2014) of evaluations in South Asia (Indore, Jabalpur, Karnal, Ludhiana, New Delhi, Ugar, Varanasi in India, Dinajpur and Jessore in Bangladesh, Bhairahawa in Nepal and Faisalabad in Pakistan).

**Fig. 2:**
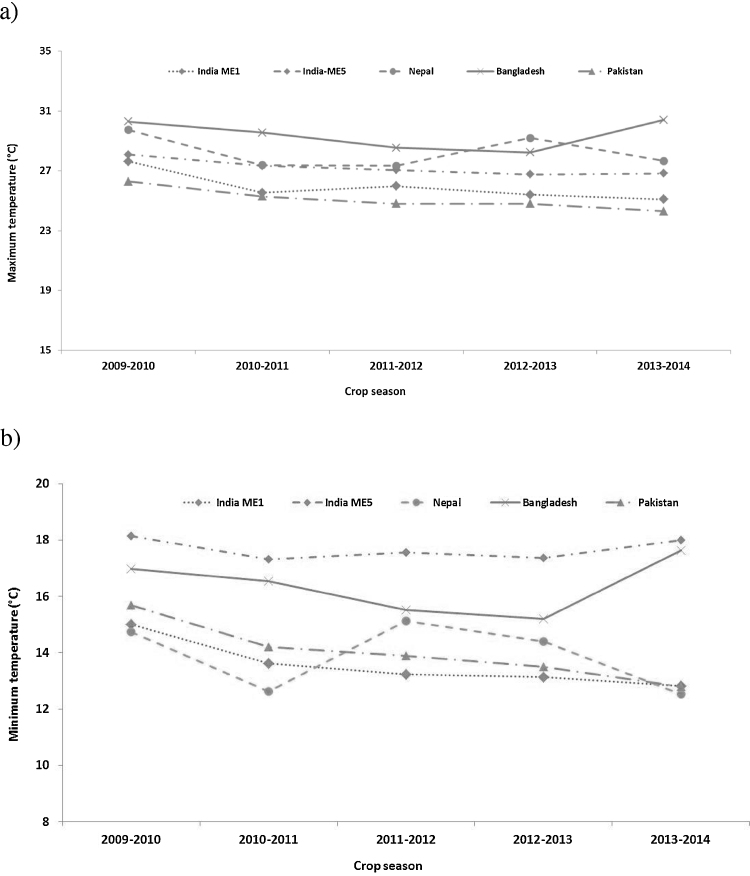
The mean a) maximum and b) minimum temperatures during the crop season from November–April in five years (2009–2014) of evaluations in India ME1 (Delhi, Karnal, Ludhiana) and India ME5 (Indore, Jabalpur, Ugar and Varanasi), Bangladesh (Dinajpur and Jessore) Nepal (Bhairahawa) and Pakistan (Faisalabad).

Mean monthly weather data from sowing till harvest in South Asia are presented in Fig. 1. The mean temperature trend is similar in all five years, with the coolest temperatures in January followed by a gradual increase till April. In the 2009–2010 crop season, mean temperatures were higher during March and April, corresponding to grain filling period than in other years. The 2011–2012 crop season had high mean monthly temperatures from November till January. Weather data for maximum and minimum temperatures were available for the following locations from 2009 to 2014: Ludhiana, Karnal, New Delhi, Varanasi and Ugar in India, Jessore and Dinajpur in Bangladesh, Bhairahawa in Nepal and Faisalabad in Pakistan. The mean maximum and minimum temperatures for Karnal, New Delhi and Ludhiana grouped as India ME1, Varanasi and Ugar grouped as India ME5, Jessore and Dinajpur in Bangladesh, and Bhairahawa in Nepal are presented in Fig. 2. Across all locations, the mean maximum temperatures in ME5 was higher by 2–3 °C than the mean maximum temperature in ME1, with the exception of 2009–2010, which was a relatively warm year in north western India and temperatures were nearly same in ME1 and ME5. Within ME1, the mean maximum temperatures in Pakistan were lower or similar to those for India ME1 across years, whereas the mean minimum temperatures in Pakistan were relatively higher. Bangladesh had the highest mean maximum temperatures, followed by Nepal and IndiaME5. The mean minimum temperatures in IndiaME5 were 1–3 °C higher than other locations across all years. Between the MEs in India there was a 4 °C difference in minimum temperatures, ME5 locations being warmer. Though classified as ME5, Bhairahawa in Nepal had cooler mean minimum temperatures compared to other ME5 locations in South Asia except, 2011–2012 and 2012–2013, when mean minimum temperatures was 2 °C higher compared to means for other crop seasons in Bhairahawa.

### 3.2 Grain yield and agronomic traits for CSISA-HT-EM trials

The DTH and PH for the CSISA-HT-EM trials were recorded at all locations and years. The mean DTH ranged from 63–111 days in South Asia and 89–111 days and 63–74 days in ME1 and ME5 respectively. The cooler ME1 locations had longer DTH compared to ME5 with a mean difference of ≥20 days (Table 2). Data for DTM were received only in some years from certain locations. A mean difference of ≥20 days was observed between the ME1 and ME5 locations for DTM. Estimated grain filling duration ranged from 25 to 30 days for ME5 to 40 days for ME1 in South Asia. The mean PH ranged from 83 to 111 cm in South Asia. On an average, PH of trials reduced by 20 cm in ME5 compared to ME1.

Mean GY varied across locations and MEs for the CSISA-HT-EM trials. The mean GY of the trials ranged from 4.13–4.28 t/ha across years (Table 3). A significant genotype and genotype-by-environment interaction variance were observed. The genotypic variance was higher than the genotype-by-environment variance in all trials. Mean GY across years was 1–2 t/ha higher for trials in ME1 locations than ME5 (Table 2). A similar difference was observed between ME1 and ME5 locations in India. Within ME5 and across South Asia, Nepal had the lowest mean GY of the trials in all years of evaluations. Differences are also observed in mean GY within ME1, with IndiaME1 having the higher grain yields than Pakistan.

**Fig. 3:**
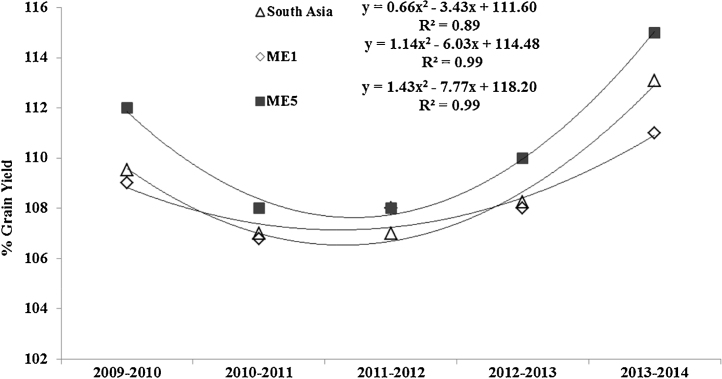
Regression of percent mean grain yield of the top five highest yielding lines in the CSISA-HT-EM trials (% grain yield) compared to local checks from 2009 to 2014 in ME1, ME 5 and across South Asia.

Each of the CSISA-HT-EM trials had wheat lines with significant higher GY than local checks. The top five highest yielding lines in each trial are listed in Table 3. The estimated Dunnett’s one tailed test statistics (at 0.05 the test statistics values were 0.41, 0.29, 0.29, 0.26, 0.65 in 2009–2010, 2010–2011, 2011–2012, 2012–2013, and 2013–2014 crop season respectively) is a conservative test and though it identified lines with significantly higher grain yield than the local checks we used Fisher’s LSD to identify the HYLs in each year of the CSISA-HT-EM trials (Table 3). The highest yielding wheat lines had a 4–10% higher grain yield than the local checks. The heritability for GY ranged from 0.52 to 0.67 across locations during 2009–2014 (Table 3). A regression analysis of% GY of the HYL lines compared to local checks showed definite positive trends in progress in over years (Fig. 3). A linear regression model estimated breeding gains of 0.52%, 0.80% and 0.83% in ME1, ME5 and South Asia respectively, though the R^2^ values were not significant. On further analysis, the progress in GY over five years fitted well in a quadratic model: y = 1.14 x^2^ − 6.03x + 114.48 (R^2^ = 0.99, p < 0.01), y = 1.43 x^2^ − 7.77x + 118.20 (R^2^ = 0.99, p < 0.01), and y = 0.66 x^2^ − 3.43 + 111.60 (R^2^ = 0.89, p < 0.01) in ME1, ME5 and South Asia respectively.

### 3.3 Association of grain yield with temperatures and days to heading

**Fig. 4:**
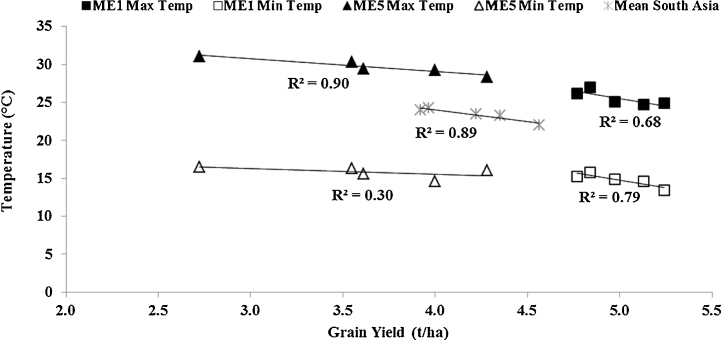
Association of mean temperatures during grain filling with mean grain yield of the CSISA-HT-EM trials across South Asia (Locations included are: Indore, Jabalpur, Karnal, Ludhiana, New Delhi, Ugar, Varanasi in India, Dinajpur and Jessore in Bangladesh, Bhairahawa in Nepal and Faisalabad in Pakistan), ME1 (Karnal, Ludhiana, New Delhi in India and Faisalabad in Pakistan) and ME5 (Indore, Jabalpur, Ugar, and Varanasi in India, Dinajpur and Jessore in Bangladesh, Bhairahawa in Nepal) from 2009 to 2014.

The mean temperatures across crop season had a negative association with mean GY (R^2^ = 0.74, p < 0.05). The crop season was grouped into pre-heading and grain filling time periods based on days to heading for the locations with temperature data. The temperatures at grain filling showed significant negative association with GY (R^2^ = 0.89, p < 0.05) compared to mean temperatures at pre-heading (Fig. 4). A negative association implies that increased temperatures reduced mean GY. Further investigation showed that the mean minimum temperatures in ME1 (R^2^ = 0.79, p < 0.05) and the mean maximum temperatures in ME5 (R^2^ = 0.90, p < 0.01) had a significant negative association with GY.

Strong genetic correlations (ranging from 0.43 to 0.79) were observed between DTH and GY across years in South Asia and significant negative association (ranging from 0.52-0.81) were estimated in ME5 (Table 4). A similar negative association of GY with DTH was observed in ME1, except for 2012–2013, where the correlation was negative but not significant.

## 4 Discussion

The CSISA-HT-EM trials were evaluated at major wheat producing areas in South Asia that represented the diverse temperature ranges in which wheat is grown in these regions. The ME classification system developed at CIMMYT enables grouping of diverse wheat growing areas in the world and helps to target breeding activities. The early-maturing high-yielding wheat lines were targeted for adaptation under terminal and continual high temperature stress in ME1 and ME5, respectively. Information on irrigation was not available for all locations but nearly all locations in ME1 and ME5 were probably irrigated. Previous studies have shown that the performance of normally sown and optimally irrigated trials in Cd. Obregon, Mexico was able to predict the performance of the genotypes in ME1 testing locations globally (Trethowan and Crossa, 2007). Likewise, the performance of the genotypes in late-sown trials under high temperature stress at Cd. Obregon was comparable to that of genotypes in ME5 locations in South Asia (Lillemo et al., 2005, Mondal et al., 2013). The breeding program at CIMMYT evaluates the advanced lines for 2 years, first year in irrigated normal sown and second year in irrigated normal and late sown for heat stress in Cd. Obregon. Early maturing lines that have stable GY in both years were selected in current study to evaluate their adaptation in South Asia.

Cropping season temperature variation had an impact on mean GY. The mean temperatures in South Asia at grain filling in 2009–2010 were higher than for other years. Such a trend has been reported by USDA (2014), where the temperatures in 2009–2010 were reported to be warmer and since 2010 the focus on higher productivity and favorable climate conditions has led to increased wheat production in South Asia. While average temperatures across crop season showed a significant negative association with GY, it was interesting to observe the impact of high temperatures at grain filling on GY in both MEs. Impact of high temperatures at grain filling in wheat has been reported in South Asia and globally (Chatrath et al., 2007, Mason et al., 2013, Zarei et al., 2013, Asseng et al., 2015). A difference of more than 1 t/ha is observed between the MEs in each year in South Asia. Similar grain yield differences between the MEs have been reported in other international trials conducted in South Asia (Sharma et al., 2012, Mondal et al., 2013, Pask et al., 2014). Within ME1, differences in GY were observed between India and Pakistan, which may have been due to temperatures or agronomic and management practices. It was observed that the Pakistan site had cooler mean maximum temperatures than those for India ME1, though the mean minimum temperatures were around 1 °C higher, with the exception of 2013–2014. In the crop season 2013–2014, the mean minimum temperatures in India ME1 and Pakistan were similar. With no reports on diseases the differences in GY may be due to local agronomic or management practices. The ME5 locations in India had relatively higher GY than locations in Nepal and Bangladesh. The maximum temperatures in ME5 locations in India were lower than those of Bangladesh, though the minimum temperatures were higher than Nepal or Bangladesh. Lower night-time temperatures are critical for wheat and studies have suggested that higher average minimum temperatures increases respiration rates in wheat and rice resulting in a negative impact on grain yield in wheat and rice (Peng et al., 2004, Pask et al., 2014). Whereas higher average minimum temperatures may have resulted in reduction of GY in Pakistan, no such effect was observed in ME5 locations in India, which may be due to the availability of irrigation and lower maximum temperatures during the crop season (than Nepal or Bangladesh). Since all locations are irrigated, delayed sowing in both Nepal and Bangladesh (which often occurs in wheat, following a rice crop) may have exposed the trial to extreme heat and thereby reducing GY. Considering the locations with similar sowing dates the average reduction of 8% in GY was estimated for a 1 °C rise in temperature.

Continual high temperatures in ME5 locations led to early heading and shorter crop duration than for ME1 sites. Previous studies have reported similar effects of high temperature stress on days to heading and maturity (Mason et al., 2010, Yang et al., 2002, Mondal et al., 2013). The observed reductions in plant height due to high temperatures were similar to those reported in other studies (Zhong-hu and Rajaram, 1994, Mondal et al., 2013). The genotypes included in the CSISA-HT-EM trials had earlier heading than local checks, which were locally-adapted high-yielding varieties with early to normal maturity. A significant negative association between DTH and GY was observed in each year implying that early lines had higher grain yields. The negative associations are significant in both terminal high temperature stress conditions in ME1 and continual high temperature stress conditions in ME5. Such a negative association has been reported in previous studies and supports the concept that earliness enables adaptation to high temperature stress (Tewolde et al., 2006, Mondal et al., 2013, Pask et al., 2014). A molecular marker analysis of the wheat lines for known *Ppd*, *Vrn* and *eps* genes showed no significant variation between the lines (Personal comm. Susanne Dreisigacker, data not shown). Further investigation is underway to identify genes that could link earliness and heat adaptation.

Several wheat lines were identified each year that outperformed the local checks. The high yielding lines were not re-evaluated in the following years as a part of the project; though the highest yielding lines were selected by the National partners for further evaluations. The estimated progress in breeding for early maturing wheat in 5 years fits a quadratic model: implying that there are two phases in the curve. Similar results were observed in by Rodrigues et al. (2007) for estimating genetic gains in wheat in Brazil. A quadratic model indicated two phases in the grain yield progress over 40 years in Brazil and was attributed to changes in breeding objectives across several decades. In this study, however, temperatures during the crop cycle may have influenced the trends observed. The biggest difference (10%) in grain yield of HYLs over local checks was in 2009–2010, the warmest crop season. The lack of adaptation of the local checks was likely the reason for the difference in GY. As average temperatures reduced in 2010–2011, the grain yields of HYLs over local checks decreased to 7% though still statistically significant. Over the subsequent years, the mean difference in GY between the HYLs and local checks increased, implying an overall positive trend in breeding for the early- maturing wheat. It also suggests the wider adaptability of the HYLs for different temperature ranges across years. While linear increases in genetic gains cud not be estimated from this study, annual gains in yield through breeding at CIMMYT have been reported to range from 0.5% to 1.1% across a ten years or more (Trethowan et al., 2002, Lopes et al., 2012, Sharma et al., 2012).

Results of the CSISA-HT-EM trials demonstrate that early-maturing, high-yielding, heat-tolerant wheat lines with good adaptation potential, developed by CIMMYT’s targeted breeding, are out-performing currently grown check varieties across MEs in South Asia. Results also suggest that earliness could be a key criterion in breeding for high temperature stress tolerance in South Asia. Short-duration wheat varieties are often preferred by farmers for use in rotation with other crops. They also require fewer inputs, especially for irrigation, due to the shorter crop cycle. Two lines from the CSISA-HT-EM trials were released in India and some are in the advanced evaluation phase with the national cooperators.

## Figures and Tables

**Table 1:** Information on the number of locations, range for planting and harvest dates and plot area across locations in the CSISA-HT-EM trials from 2009 to 2014 in South Asia (Detailed information in Supplementary Table 1).

Year	No. of Locations	Planting date	Harvest Date	Plot area (m^2^)
2009–2010	9	04Dec–19Dec, 2009	30Mar–10May, 2010	2.4–6.9
2010–2011	10	10Nov–29Dec, 2010	23Mar–09May, 2011	3.0–8.3
2011–2012	11	04Nov–28Dec, 2011	22Mar–10May, 2012	2.8–8.1
2012–2013	12	04Nov–25Dec,2012	24Mar–16May, 2013	2.8–9.0
2013–2014	13	09Nov–26Dec, 2013	15Mar–17May,2014	1.5–8.3

**Table 2:** Country wise mean grain yield, days to heading, days to maturity and plant height in the CSISA-HT-EM trials from 2009 to 2014 in South Asia.

Year	Country/Region	ME	No. of Locations	Grain Yield (t/ha)	Days to Heading (days)	Days to Maturity (days)	Plant Height (cm)
2009–2010	India	ME1	3	4.96	89	–	94
		ME5	3	3.90	64	–	75
	Pakistan	ME1	1	4.72	89	–	96
	Nepal	ME5	1	2.52	68	–	83
	Bangladesh	ME5	1	4.42	71	104	100

2010–2011	India	ME1	5	5.17	96	–	108
		ME5	5	3.62	70	–	100
	Pakistan	ME1	1	4.52	104	–	–
	Nepal	ME5	1	2.33	68	–	–
	Bangladesh	ME5	1	4.52	68	97	102

2011–2012	India	ME1	3	5.72	92	–	108
		ME5	8	4.00	70	–	101
	Pakistan	ME1	1	4.61	111	–	111
	Nepal	ME5	1	1.77	74	96	77
	Bangladesh	ME5	2	3.50	67	–	96

2012–2013	India	ME1	5	5.13	96	–	92
		ME5	8	3.76	68	–	90
	Pakistan	ME1	1	5.26	97	–	–
	Nepal	ME5	1	2.28	70	–	84
	Bangladesh	ME5	2	3.69	65	–	94

2013–2014	India	ME1	5	5.45	92	–	97
		ME5	8	4.34	63	103	92
	Pakistan	ME1	1	4.72	101	141	104
	Nepal	ME5	1	2.88	71	98	87
	Bangladesh	ME5	2	4.22	64	99	95

**Table 3:** Mean grain yield (GY, t/ha), percent grain yield (%GY) compared to local checks (LC)and days to heading (DTH) for the top five highest yielding lines in the CSISA-HT-EM trials in five years of testing (2009–2014) across South Asia.

Year	GID	Entry No.	Pedigree	GY	%GY(LC)	DTH
2009–2010						
	5552006	5	HUW234 + LR34/PRINIA//KRONSTAD F2004	4.63	112	74
	5794480	27	WAXWING//INQALAB 91*2/KUKUNA/3/WBLL1….	4.55	110	74
	5398434	3	FRANCOLIN #1	4.53	109	74
	5390612	4	SUPER152	4.47	108	75
	5792819	13	WAXWING*2/CIRCUS	4.44	107	77
			**Local checks**	4.13		78
			**Trial mean**	4.26		76
			**Fisher’s LSD (at 0.05)**	0.28		0.72
			**Heritability**	0.67		0.87

2010–2011						
	5994247	25	HUW234 + LR34/PRINIA*2//KIRITATI	4.68	110	76
	5995318	11	FRET2*2/4/SNI/TRAP#1/3/KAUZ*2/TRAP//KAUZ⋯.	4.51	106	80
	5995481	27	HUW234 + LR34/PRINIA*2//KIRITATI	4.48	105	76
	5994249	8	WBLL1/KUKUNA//TACUPETO F2001/5/BAJ	4.47	105	79
	5993822	16	FRET2*2/KUKUNA//PVN/5/FRET2*2/4/SNI/TRAP#1.	4.45	105	78
			**Local checks**	4.24		81
			**Trial mean**	4.28		78
			**Fisher’s LSD (at 0.05)**	0.20		0.63
			**Heritability**	0.64		0.84

2011–2012						
	6178973	12	PFAU/SERI.1B//AMAD/3/WAXWING/4/BABAX/….	4.34	107	76
	6176225	5	FRET2/TUKURU//FRET2/3/MUNIA/CHTO//AMSEL.	4.27	105	77
	6174889	14	BECARD/KACHU	4.27	105	76
	6177554	21	WAXWING/4/SNI/TRAP#1/3/KAUZ*2/TRAP//KAUZ.	4.25	104	74
	6177851	11	PARUS/FRANCOLIN #1	4.23	104	76
			**Local checks**	4.07		77
			**Trial mean**	4.15		76
			**Fisher’s LSD (at 0.05)**	0.20		0.63
			**Heritability**	0.58		0.90

2012–2013						
	6338916	18	BAJ #1*2/HUIRIVIS #1	4.42	110	75
	6415882	27	KAUZ/PASTOR//PBW343/3/KIRITATI/4/FRNCLN	4.39	109	76
	6417076	24	BAJ #1/PAURAQ	4.34	108	74
	6337327	17	SUP152/AKURI//SUP152	4.32	107	77
	6416509	29	ND643/2*WBLL1//2*BAJ #1	4.31	107	76
			**Local checks**	4.03		75
			**Trial mean**	4.23		75
			**Fisher’s LSD (at 0.05)**	0.18		0.67
			**Heritability**	0.52		0.76

2013–2014						
	6568291	13	BAJ #1/SUP152	4.60	116	76
	6681464	28	FRANCOLIN #1*2//ND643/2*WBLL1	4.53	114	75
	6684197	18	MUTUS*2//ND643/2*WBLL1	4.44	112	79
	6568165	12	SUP152/FRNCLN	4.41	111	76
	6684208	20	FRNCLN*2//TAM200/TUI	4.41	111	76
			**Local checks**	3.96		78
			**Trial mean**	4.19		77
			**Fisher’s LSD (at 0.05)**	0.47		1.4
			**Heritability**	0.65		0.77

**Table 4:** Genetic correlations between grain yield and days to heading in ME1, ME5 and across South Asia from 2009 to 2014.

Year	ME1	ME5	South Asia
2009–2010	−0.51^**^	−0.81^***^	−0.79^***^
2010–2011	−0.52^**^	−0.74^***^	−0.73^***^
2011–2012	−0.56^**^	−0.74^***^	−0.65^**^
2012–2013	−0.39 ns^a^	−0.52^*^	−0.43^*^
2013–2014	−0.42^*^	−0.77^***^	−0.65^**^

^*^Significance at 0.01 probability level, ^**^Significance at 0.05 probability level.

^***^Significance at 0.001 probability level.

a ns-nonsignificant.
