# Supplementary material for: Grain yield, adaptation and progress in breeding for early-maturing and heat-tolerant wheat lines in South Asia
Source: Field Crops Res. 2016 Jun;192:78–85. doi: 10.1016/j.fcr.2016.04.017 (PMC4892352; doi:10.1016/j.fcr.2016.04.017)
Supplement: Supplementary file 1 [file mmc1.docx]

**Supplementary Table 1**

**Information on latitude, longitude, planting date, harvest date, plot area and irrigation for all locations in CSISA-HT-EM trials from 2009-2014 in South Asia**

| **Year** | **Country** | **Location** | **Latitude** | **Longitude** | **ME** | **Planting Date** | **Harvest Date** | **Plot Area (m^2^)** | **Irrigation** |
| --- | --- | --- | --- | --- | --- | --- | --- | --- | --- |
| **2009-2010** | Bangladesh | Dinajpur | 25°38´N | 88°41´E | ME5 | 17/12/2009 | 08/04/2010 | 5.0 | Yes |
|  | India | Karnal | 29°43´N | 75°57´E | ME1 | 15/12/2009 | 25/04/2010 | 5.4 | - |
|  | India | Indore | 22°37´N | 75°50´E | ME5 | - | - | 6.0 | Yes |
|  | India | Ludhiana | 30°54´N | 75°48´E | ME1 | 04/12/2009 | 10/05/2009 | 5.5 | Yes |
|  | India | New Delhi | 28°35'N | 77°12'E | ME1 | 15/12/2009 | 25/04/2010 | 6.9 | - |
|  | India | Ugar | 16°65'N | 74°81'E | ME5 | 29/12/2009 | 30/03/2010 | 4.6 | Yes |
|  | India | Varanasi | 25°26´N | 82°98´E | ME5 | 25/12/2009 | 10/05/2009 | 2.4 | - |
|  | Nepal | Bhairahawa | 27°32´N | 83°25´E | ME5 | 24/12/2009 | 21/04/2010 | 3.0 | - |
|  | Pakistan | Faisalabad | 31°25´N | 73°09´E | ME1 | 11/12/2009 | 21/04/2009 | 8.1 | Yes |
|  |  |  |  |  |  |  |  |  |  |
| **2010-2011** | Bangladesh | Dinajpur | 25°38´N | 88°41´E | ME5 | 20/12/2010 | 15/04/2011 | 4.0 | Yes |
|  | India | Gurgaon | 28°37´N | 77°04´E | ME1 | 10/11/2010 | 15/04/2011 | 8.3 | Yes |
|  | India | Indore | 22°37´N | 75°50´E | ME5 | 12/12/2010 | 27/04/2011 | 4.6 | Yes |
|  | India | Jalandhar | 30°54´N | 75°48´E | ME1 | 19/11/2010 | - | 7.8 | - |
|  | India | Jalna | 19°51´N | 75°53´E | ME5 | 26/11/2010 | 15/04/2011 | 3.7 | Yes |
|  | India | Karnal | 29°43´N | 75°57´E | ME1 | 19/12/2010 | 26/04/2011 | 5.5 | - |
|  | India | Ludhiana | 30°54´N | 75°48´E | ME1 | 17/11/2010 | 29/04/2011 | 6.2 | Yes |
|  | India | New Delhi | 28°35'N | 77°12'E | ME1 | 15/12/2010 | - | 6.9 | - |
|  | India | Ugar | 16°65'N | 74°81'E | ME5 | 22/12/2010 | 10/04/2011 | 3.7 | Yes |
|  | India | Varanasi | 25°26´N | 82°98´E | ME5 | 09/12/2010 | 09/05/2011 | 3.6 | Yes |
|  | India | Vijapur | 23°35´N | 72°45´E | ME5 | 16/11/2010 | 23/03/2011 | 3.5 | - |
|  | Nepal | Bhairahawa | 27°32´N | 83°25´E | ME5 | 29/12/2010 | 03/04/2011 | 3.0 | - |
|  | Pakistan | Faisalabad | 31°25´N | 73°09´E | ME1 | 29/11/2010 | 19/04/2011 | 8.1 | Yes |
|  |  |  |  |  |  |  |  |  |  |
| **2011-2012** | Bangladesh | Dinajpur | 25°38´N | 88°41´E | ME5 | - | 15/04/2012 | 4.0 |  |
|  | Bangladesh | Jessore | 23°11'N | 89°14'E | ME5 | 22/11/2011 | 01/04/2012 | 3.2 | Yes |
|  | India | Ghajipur | 26°47'N | 82°12'E | ME5 | 12/12/2011 | 03/05/2012 | 2.3 | - |
|  | India | Indore | 22°37'N | 75°50'E | ME1 | 09/12/2011 | 29/04/2012 | 4.6 | Yes |
|  | India | Jalna | 19°51'N | 75°53'E | ME5 | 04/11/2011 | 22/03/2012 | 5.5 | Yes |
|  | India | Kanpur | 26°28'N | 80°24'E | ME5 | 16/12/2011 | 16/04/2012 | 3.8 | Yes |
|  | India | Karnal | 29°40'N | 77°02'E | ME1 | 15/12/2011 | 01/05/2012 | 4.3 | - |
|  | India | Ludhiana | 30°56'N | 75°52'E | ME1 | 19/11/2011 | 10/05/2012 | 5.5 | Yes |
|  | India | New Delhi | 28°35'N | 77°12'E | ME1 | 05/12/2011 | 20/04/2012 | 6.9 | Yes |
|  | India | Niphad | 20°6'N | 74°6'E | ME5 | 25/11/2011 | 31/03/2012 | - | Yes |
|  | India | Ugar | 16°65'N | 74°81'E | ME5 | 10/12/2011 | 31/03/2012 | 3.7 | Yes |
|  | India | Varanasi | 25°18'N | 83°30'E | ME5 | 09/12/2011 | 25/04/2012 | 2.8 | Yes |
|  | India | Vijapur | 23°35'N | 72°45'E | ME5 | 25/11/2011 | 03/05/2012 | 3.5 | Yes |
|  | Nepal | Bhairahawa | 27°32´N | 83°25´E | ME5 | 28/12/2011 | 03/05/3012 | 3.0 | - |
|  | Pakistan | Faisalabad | 31°25´N | 73°09´E | ME1 | 24/11/2011 | 19/04/2012 | 8.1 | Yes |
|  |  |  |  |  |  |  |  |  |  |
| **2012-2013** | Bangladesh | Dinajpur | 25°38´N | 88°41´E | ME5 | 20/12/2012 | 09/04/2013 | 4.0 | Yes |
|  | Bangladesh | Jessore | 23°11'N | 89°14'E | ME5 | 23/11/2012 | 15/04/2013 | 4.0 | Yes |
|  | India | Ghajipur | 26°47'N | 82°12'E | ME5 | 20/12/2012 | 06/05/2013 | 2.3 | Yes |
|  | India | Gurgaon | 28°37´N | 77°04´E | ME1 | 08/11/2012 | 10/04/2013 | 8.3 | Yes |
|  | India | Indore | 22°37'N | 75°50'E | ME5 | 10/12/2012 | 16/05/2013 | 3.6 | Yes |
|  | India | Jabalpur | 23°09'N | 79°58'E | ME5 | 25/11/2012 | 08/04/2013 | 3.0 | Yes |
|  | India | Jalandhar | 30°54'N | 75°48'E | ME1 | 17/11/2012 | 27/04/2013 | 8.3 | Yes |
|  | India | Jalna | 19°51'N | 75°53'E | ME5 | 29/11/2012 | 16/04/2013 | 3.7 | Yes |
|  | India | Kanpur | 26°28'N | 80°24'E | ME5 | 30/11/2012 | 01/05/2013 | 6.0 | Yes |
|  | India | Ludhiana | 30°56'N | 75°52'E | ME1 | 21/11/2012 | 02/05/2013 | 6.0 | Yes |
|  | India | Karnal | 29°40'N | 77°02'E | ME1 | 20/12/2013 | 21/04/2014 | 4.8 | Yes |
|  | India | New Delhi | 28°35'N | 77°12'E | ME1 | 13/12/2012 | 28/04/2013 | 6.5 | Yes |
|  | India | Niphad | 20°6'N | 74°6'E | ME5 | 11/11/2012 | 24/03/2013 | - | Yes |
|  | India | Ugar | 16°65'N | 74°81'E | ME5 | 07/12/2012 | 01/04/2013 | 4.0 | Yes |
|  | India | Varanasi | 25°18'N | 83°30'E | ME5 | 08/12/2012 | 17/04/2013 | 2.8 | Yes |
|  | Nepal | Bhairahawa | 27°30'N | 83°27'E | ME5 | 25/12/2012 | 03/04/2013 | 3.0 | Yes |
|  | Pakistan | Faisalabad | 31°25´N | 73°09´E | ME1 | 23/11/2012 | 08/05/2013 | 9.0 | Yes |
|  |  |  |  |  |  |  |  |  |  |
| **2013-2014** | Bangladesh | Dinajpur | 25°38´N | 88°41´E | ME5 | 25/12/2013 | 17/04/2014 | 6.0 | Yes |
|  | Bangladesh | Jessore | 23°11'N | 89°14'E | ME5 | 22/12/2013 | 06/04/2014 | 4.0 | Yes |
|  | India | Ugar | 16°65'N | 74°81'E | ME5 | 07/12/2013 | 26/03/2014 | 3.2 | Yes |
|  | India | Gangapur | 19°53'N | 72°23'E | ME5 | 25/11/2013 | 11/03/2014 | 3.7 | Yes |
|  | India | Gurgaon | 28°37´N | 77°04´E | ME1 | 09/11/2013 | 05/04/2014 | 8.3 | Yes |
|  | India | Indore | 22°37'N | 75°50'E | ME5 | 10/12/2013 | 17/05/2014 | 3.6 | Yes |
|  | India | Indore | 22°37'N | 75°50'E | ME5 | 25/11/2013 | 05/04/2014 | 6.9 | Yes |
|  | India | Jalandhar | 30°54'N | 75°48'E | ME1 | 08/11/2013 | 07/05/2014 | 8.3 | Yes |
|  | India | Jalna | 19°51'N | 75°53'E | ME5 | 02/12/2013 | 18/04/2014 | 3.7 | Yes |
|  | India | Karnal | 29°40'N | 77°02'E | ME1 | 20/12/2013 | 21/04/2014 | 4.8 | Yes |
|  | India | Ludhiana | 30°56'N | 75°52'E | ME1 | 09/11/2013 | 05/05/2014 | 6.0 | Yes |
|  | India | Malda | 25°2'N | 88°6'E | ME5 | 09/11/2013 | 01/04/2014 | 4.0 | Yes |
|  | India | New Delhi | 28°35'N | 77°12'E | ME1 | 15/12/2013 | 29/04/2014 | 7.2 | Yes |
|  | India | Niphad | 20°6'N | 74°6'E | ME5 | 17/11/2013 | 15/03/2014 | 1.5 | Yes |
|  | India | Varanasi | 25°18'N | 83°30'E | ME5 | 08/12/2013 | 20/04/2014 | 2.4 | Yes |
|  | Nepal | Bhairahawa | 27°30'N | 83°27'E | ME5 | 26/12/2013 | 05/05/2014 | 3.0 | Yes |
|  | Pakistan | Faisalabad | 31°25´N | 73°09´E | ME1 | 21/11/2014 | 09/05/2014 | 8.1 | Yes |
|  |  |  |  |  |  |  |  |  |  |
